# Environmental DNA analysis confirms extant populations of the cryptic Irwin’s turtle within its historical range

**DOI:** 10.1186/s12862-022-02009-6

**Published:** 2022-05-02

**Authors:** Cecilia Villacorta-Rath, Thomas Espinoza, Bernie Cockayne, Jason Schaffer, Damien Burrows

**Affiliations:** 1 Centre for Tropical Water and Aquatic Ecosystem Research (TropWATER), James Cook University, Townsville, QLD 4811 Australia; 2 Department of Regional Development, Manufacturing and Water, Bundaberg, QLD Australia; 3 Reef Catchments (Mackay Whitsunday Isaac) Limited, Mackay, QLD Australia

**Keywords:** Catchment-wide survey, Dam development, *Elseya irwini*, eDNA, Monitoring, User-friendly field methods

## Abstract

**Background:**

Approximately 50% of freshwater turtles worldwide are currently threatened by habitat loss, rural development and altered stream flows. Paradoxically, reptiles are understudied organisms, with many species lacking basic geographic distribution and abundance data. The iconic Irwin’s turtle, *Elseya irwini*, belongs to a unique group of Australian endemic freshwater turtles capable of cloacal respiration. Water resource development, increased presence of saltwater crocodiles and its cryptic behaviour, have made sampling for Irwin’s turtle in parts of its range problematic, resulting in no confirmed detections across much of its known range for > 25 years. Here, we used environmental DNA (eDNA) analysis for *E. irwini* detection along its historical and contemporary distribution in the Burdekin, Bowen and Broken River catchments and tributaries. Five replicate water samples were collected at 37 sites across those three river catchments. Environmental DNA was extracted using a glycogen-aided precipitation method and screened for the presence of *E. irwini* through an eDNA assay targeting a 127 base pair-long fragment of the NADH dehydrogenase 4 (ND4) mitochondrial gene.

**Results:**

*Elseya irwini* eDNA was detected at sites within its historic distribution in the lower Burdekin River, where the species had not been formally recorded for > 25 years, indicating the species still inhabits the lower Burdekin area. We also found higher levels of *E. iriwni* eDNA within its contemporary distribution in the Bowen and Broken Rivers, matching the prevailing scientific view that these areas host larger populations of *E. irwini*.

**Conclusions:**

This study constitutes the first scientific evidence of *E. irwini* presence in the lower Burdekin since the original type specimens were collected as part of its formal description, shortly after the construction of the Burdekin Falls Dam. From the higher percentage of positive detections in the upper reaches of the Broken River (Urannah Creek), we conclude that this area constitutes the core habitat area for the species. Our field protocol comprises a user-friendly, time-effective sampling method. Finally, due to safety risks associated with traditional turtle sampling methods in the Burdekin River (e.g., estuarine crocodiles) we propose eDNA sampling as the most pragmatic detection method available for *E. irwini*.

**Supplementary Information:**

The online version contains supplementary material available at 10.1186/s12862-022-02009-6.

## Background

Conservation of the world’s reptiles is impeded by a lack of information relating to distribution, systematics and ecology [1, 2]. In addition, almost half of global turtle and tortoise species are threatened by anthropogenic effects such as habitat loss, rural development and altered streamflow regimes [3]. In Australia, the Chelidae family includes a particular group of freshwater turtles of high conservation value in the genus *Elseya* that can supplement respiration via diffusion across the vascularised surface of their cloaca [4, 5]. One of the main threats to this group of freshwater turtles is riverine development and in particular, water impoundment [6–10], which alters their habitat as well as natural flow regimes and water quality, affecting dissolved oxygen availability and increased sedimentation within and downstream of impoundment structures [11–13]. For example, weir development along the Fitzroy River catchment, central Queensland, has altered *Elseya albagula*’s nesting grounds, directly impacting recruitment of the species [6, 14]. The *Elseya* species in the adjoining Burdekin catchment (*E. irwini*), occurs in the lower Burdekin River, the Bowen River (a major tributary of the Burdekin River) and in various upland tributaries of the Bowen River such as the Broken River and Urannah and Massey creeks [15–17]. Its habitat in the lower Burdekin River has been subject to a large dam development (the Burdekin Falls Dam—completed in 1987) that changed the flow regime and greatly increased the turbidity of the lower river [18, 19]. The status of *E. irwini* in the lower Burdekin River, where they have not been formally recorded since the 1990’s ([15], Atlas of Living Australia, ala.org.au), shortly after construction of the Burdekin Falls Dam, is unknown. In addition, a proposal to build a dam in the upper reaches of the Broken Rover at Urannah Station is currently being evaluated. Given this situation, it is critical that their distribution is determined both in their historical range in the lower Burdekin River and in the upper tributaries.

Effective surveys of the lower Burdekin River for *E. irwini* have been limited because the species, due to its underwater respiratory capability, rarely surfaces; is extremely trap-shy, rarely being caught in underwater traps (TropWATER unpublished data); the presence of estuarine crocodiles prevent diving, snorkeling or other forms of manual in-water survey; and high water turbidity greatly limits visibility for in-water observations or underwater cameras [4, 17]. Similar to other cloacally respiring species, *E. irwini* prefer clear, well-oxygenated water with perennial flow for their specialised breathing [17, 20–22] whereas the Burdekin River below the Burdekin Falls Dam, which was formerly relatively clear most of the time, has remained persistently turbid since the dams construction [18, 19]. In clear waters, *E. irwini*’s dive time is positively correlated to dissolved oxygen and negatively correlated to temperature [17]. A recent study on dive duration of *E. irwini* under different water quality scenarios found that in the presence of increased suspended solids, mean dive duration is reduced by 73% under winter mean temperature and high dissolved oxygen saturation [17]. This highlights the impact that increased turbidity could have on *E. irwini* survival and persisence [17].

In contrast, the upper tributaries of the Broken River, which are considered high quality and likely refugia habitats for this species [17], have long been the subject of dam proposals, one of which at Urannah Station is currently being evaluated, have clear water, are largely free from estuarine crocodiles. Although these streams are more suitable for visual underwater census; their remoteness and absence of road access in many cases make such surveys labour intensive, difficult and expensive, such that much of the putatively suitable habitat there has never been surveyed for turtles.

Environmental DNA (eDNA) sampling is ideal for species such as *E. irwini* that are cryptic, located in remote or difficult-to-sample locations and not easily sampled using traditional methods. By targeting DNA shed into the aquatic environment, large geographic areas can be screened in short periods of time to determine species presence with high confidence [23]. Environmental DNA sampling is becoming increasingly recognised as an effective biomonitoring tool to detect species in a range of ecosystems [24]. Although eDNA shedding rates of freshwater turtles are lower than other aquatic vertebrates due to their keratinised exterior integument [25, 26], the rising number of studies focusing on freshwater turtle eDNA detection shows the potential of the technique to target rare and cryptic turtle species [25, 27–31]. In the particular case of *E. irwini*, eDNA surveys are the best available option for detection in the lower Burdekin, given the survey challenges described above. The constant flushing of water through their bursae could be a means of eDNA shedding in *Elseya* turtles, as opposed to other aquatic reptiles that have been suggested to exhibit very limited eDNA shedding [32]. Additionally, the remote location of some of the upland sites where the species is thought to inhabit increases the travel time, difficulty and, therefore, the cost of field sampling. Consequently, integrating non-specialists for sample collection (e.g., Traditional Owner groups, government agencies, consultancy companies, etc.) can enhance monitoring capability.

When engaging with non-specialists, it is crucial to have ‘user-friendly’ eDNA field protocols [33], which are concise and easy-to-follow. This reduces the risk of contamination in the field, which is one of the main sources of false positives [22] and ensures the method's reproducibility. Environmental DNA does not disperse evenly in the water column, either horizontally or vertically [34, 35]; therefore, eDNA practitioners recommend filtering large volumes of water to capture available eDNA [36]. Yet a recent study comparing a large volume sampler (> 1000 L) against precipitating eDNA from preserved whole water samples (300 mL) for detection of a Critically Endangered rainforest frog suggested that water precipitation can be as effective as filtration [37]. The main advantage of collecting and preserving whole water samples for later precipitation is its simplicity and the very small amount of required field equipment, which is important when surveying remote locations. Therefore, precipitating water from preserved samples can potentially open the opportunity of engagement with different stakeholders and end-users, making eDNA methods more accessible. For *E. irwini*, this would allow surveying a broader range sites the species potentially inhabits, providing better knowledge of its distribution, ultimately aiding conservation measures. This study, involving field sample collection by both eDNA scientists and non-eDNA-specialists, aimed to conduct a catchment-wide assessment of *E. irwini*, covering sites of historic distribution in the lower Burdekin River, and contemporary distributions in the Bowen and Broken Rivers, including sites that would be affected by proposed water resource development.

## Results

### Lower Burdekin River catchment

**Fig. 1:**
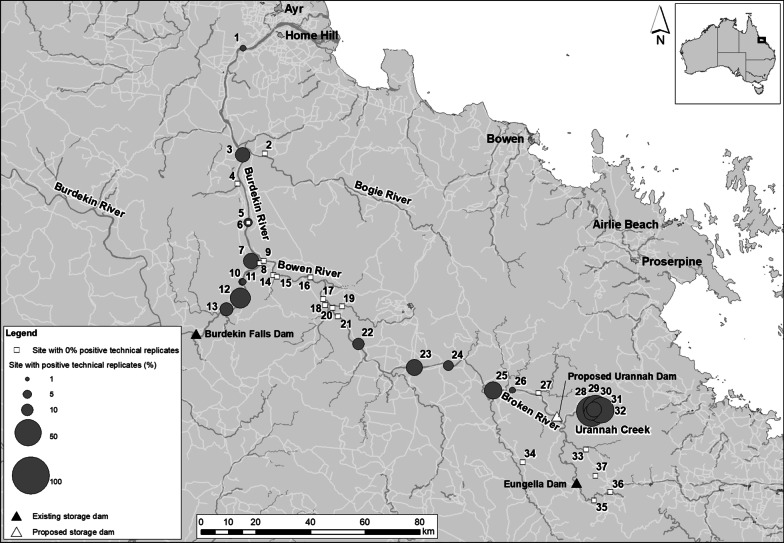
Sampling sites surveyed by three different institutions during 2020 and 2021 for *E. irwini* eDNA detection along the Burdekin, Bowen and Broken River catchments

Environmental DNA was detected at eight sites in the lower Burdekin catchment, including two sites located just 12–13.5 km downstream of the Burdekin Falls Dam. Although 80% of the biological replicates at both sites showed positive amplification (4/5 replicates), there were more qPCR technical replicates with positive detections at the more downstream site (Burdekin Falls Dam downstream 1; 12/40 replicates) than at the site 1.5 km upstream (Burdekin Falls Dam downstream 2; 5/40 replicates) (Table 1, Fig. 1). The two sites sampled at the Gorge Weir (6 km downstream from the site Burdekin Falls Dam downstream 2) also showed positive eDNA detections, however, at a very low percentage: 1/5 biological and 1/40 qPCR technical replicates at Gorge Weir 1; 1/4 biological and 1/32 qPCR technical replicates at Gorge Weir 2 (Table 1, Fig. 1). The site at the Burdekin and Bowen River junction, where the first *E. irwini* specimen came to the attention of scientists [15], showed positive eDNA detections. Although only one biological replicate at this site was positive, seven out of 40 of the technical replicates showed positive amplification (Table 1, Fig. 1). From two sites located ~ 14.5 km from the Burdekin and Bowen River junction, only one of them, Dalbeg, showed positive eDNA detections at one out of five biological replicates (Table 1, Fig. 1). However, only two out of 40 qPCR technical replicates had positive amplifications suggesting that those detections could be due to eDNA transport from upstream sites. However, an increase in eDNA detections was observed at Milllaroo, where 80% of biological replicates (4/5 replicates) and 15% qPCR technical replicates (6/40 replicates) showed positive eDNA detections (Table 1, Fig. 1). Finally, the most downstream site sampled in the lower Burdekin area, The Rocks, also showed positive eDNA detections, however, at only one out of five biological and one out of 40 qPCR technical replicates (Table 1, Fig. 1). This site was where the species’ original holotype (Queensland Museum, Q. M. J59431) was collected in 1993 [15].

**Table 1:** Detection of *E. irwini* eDNA in water samples collected along the Burdekin, Bowen and Broken River catchments

Catchment	Site	Biological replicates			Technical replicates		
		# replicates	# positive replicates	% positive replicates	# replicates	# positive replicates	% positive replicates
Burdekin	The Rocks^a^ [1]	**5**	**1**	**20**	**40**	**1**	**2.5**
	Bogie River^b^ [2]	5	0	0	40	0	0
	Millaroo^a^ [3]	**5**	**4**	**80**	**40**	**6**	**15**
	Expedition Pass Creek^b^ [4]	5	0	0	40	0	0
	Dalbeg^a^ [5]	**5**	**1**	**20**	**40**	**2**	**5**
	Johnny Cake Road^a^ [6]	5	0	0	40	0	0
	Burdekin and Bowen junction^a^ [7]	**5**	**1**	**20**	**40**	**7**	**17.5**
	Blue Valley 1^a^ [8]	5	0	0	40	0	0
	Blue Valley 2^a^ [9]	5	0	0	40	0	0
	Gorge Weir 1^a,c^ [10]	**5**	**1**	**20**	**40**	**1**	**2.5**
	Gorge Weir 2^a,c^,* (11)	**4**	**1**	**25**	**32**	**1**	**3.13**
	Burdekin Falls Dam downstream 1^a^ [12]	**5**	**4**	**80**	**40**	**12**	**30**
	Burdekin Falls Dam downstream 2^a^ [13]	**5**	**4**	**80**	**40**	**5**	**12.5**
Bowen	Terrible Creek^a^ [14]	5	0	0	40	0	0
	Terrible Creek upstream^a^ [15]	5	0	0	40	0	0
	Riverview^a^ [16]	5	0	0	40	0	0
	Bowen River Hotel^a^ [17]	5	0	0	40	0	0
	Bowen River Hotel upstream^a^ [18]	5	0	0	40	0	0
	Pelican Creek^b^ [19]	5	0	0	40	0	0
	Myuna 1^a^ [20]	5	0	0	40	0	0
	Myuna 2^a^ [21]	5	0	0	40	0	0
	Birralee^a^ [22]	**5**	**2**	**40**	**40**	**4**	**10**
	Bowen Developmental Road^a^ [23]	**5**	**4**	**80**	**40**	**8**	**20**
	Exmoor Road^a^ [24]	**5**	**3**	**60**	**40**	**3**	**7.5**
Broken	Broken River^a^ [25]	**5**	**4**	**80**	**40**	**9**	**22.5**
	Mount Sugarloaf^a^ [26]	**5**	**1**	**20**	**40**	**1**	**2.5**
	Grant Creek^b^ [27]	5	0	0	40	0	0
	Urannah Creek 1^b^ [28]	**5**	**2**	**40**	**40**	**7**	**17.5**
	Urannah Creek 2^b^ [29]	**5**	**5**	**100**	**40**	**27**	**67.5**
	Urannah Creek 3^b^ [30]	**5**	**4**	**80**	**40**	**7**	**17.5**
	Urannah Creek 4^b^ [31]	**5**	**3**	**60**	**40**	**19**	**47.5**
	Urannah Creek 5^b^ [32]	**5**	**5**	**100**	**40**	**19**	**47.5**
	Massey Gorge^b^ [33]	5	0	0	40	0	0
	Blenheim^b^ [34]	5	0	0	40	0	0
	Old Racecourse^b^ [35]	5	0	0	40	0	0
	Resort^a^ [36]	5	0	0	40	0	0
	Bee Creek^b^ [37]	5	0	0	40	0	0

Numbers in brackets next to site name indicate the site number in Fig. 1. Sites with positive detections are indicated in bold

^a^Sites located on the main river channel; ^b^sites located on a tributary; ^c^sites located on a weir; *one sample jar was compromised during transport

### Bowen River catchment

Environmental DNA detections along the Bowen River were restricted to three sites in the upper catchment: Birralee, Bowen Developmental Road and Exmoor Road (Table 1, Fig. 1). The percentage of qPCR technical replicates with positive eDNA detections at these sites were similar to those in the lower Burdekin, 7.5% at Exmoor Road (3/40 replicates), 10% at Birralee (4/40 replicates) and 20% at Bowen Developmental Road (8/40 replicates) (Table 1, Fig. 1). It is worth mentioning that the site ‘Bowen Developmental Road’ was located 9 km downstream from the Collinsville Weir wall.

### Broken River catchment

Two sites in the lower Broken River catchment showed positive eDNA detections: Broken River (4/5 biological and 9/40 qPCR techical replicates) and Mount Sugarloaf (1/5 biological and 1/40 qPCR techical replicates) (Table 1, Fig. 1). Furthermore, eDNA detections were obtained from all five sites sampled along a 5 km stretch of Urannah Creek, a major tributary of the Broken River (Table 1). The highest percentage of qPCR technical replicates with eDNA detections were observed at these sites, going from 17.5% at Urannah Creek 1 and 3 (7/40 replicates), 47.5% at Urannah Creek 4 and 5 (19/40 replicates) and 67.5% at Urannah Creek 2 (27/40 replicates) (Table 1, Fig. 1). There were no eDNA detections at Blenheim, Old Racecorse, Resort and Bee Creek (sites 34–37). This was expected as sites 34 and 35 were in tributaries with limited suitable habitat and sites 36 and 37 were above the known upstream limits of *E. irwini*. Massey Creek is expected to provide suitable habitat for *E. irwini*, but has not been sampled due to limited access. The one site we sampled (site 33), which failed to show any eDNA, was well within rainforest reaches and may have been too far upstream on that creek for *E. irwini*. Inhibition tests showed that water samples had a ΔCt ≤ 3, or just above 3, demonstrating that no false negatives due to inhibition were present in the qPCR analysis (Additional file 1: Table S1). Two samples (Gorge Weir 1 rep5 and Johnny Cake Road Control) exhibited a ΔCt > 10, therefore, a 1:10 dilution was applied to this sample to overcome inhibition and re-run on qPCR. Finally, none of the field blanks from the 37 sampling sites, extraction controls and qPCR NTCs exhibited positive eDNA amplification, proving that no contamination was introduced during sampling, eDNA extraction or qPCR analysis.

### Environmental factors affecting eDNA detectability

**Fig. 2:**
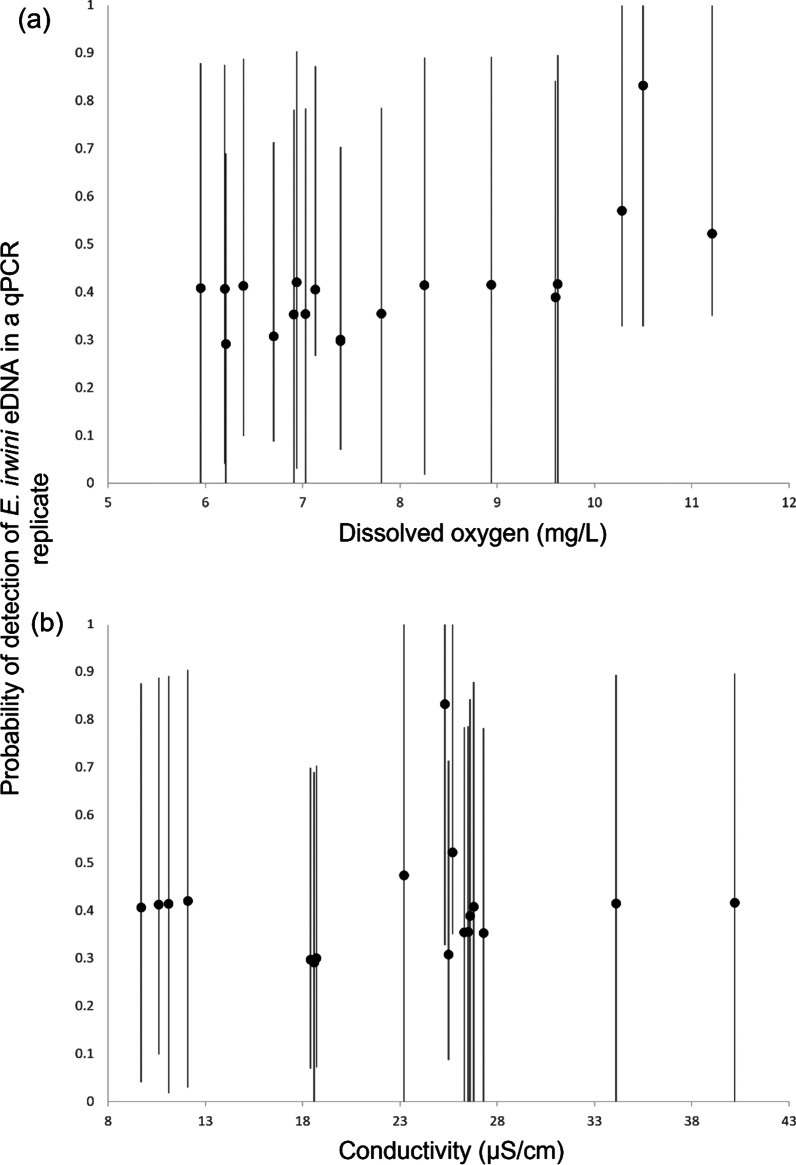
Estimated probabilities of detection of *E. irwini* eDNA in qPCR replicates. Symbols are estimates of posterior medians, with error bars indicating 95% confidence intervals

The model that best described eDNA detection data determined dissolved oxygen (mg L^−1^) as the primary explanatory variable at the site and water conductivity (µS cm^−1^) as the primary explanatory variable for biological and technical replicates (Fig. 2, Additional file 1: Table S2). However, there was a large variation in eDNA detection across sites, the 95% confidence intervals are quite large and overlapping, making it impossible to make generalizations about the probability of eDNA detection and those two environmental factors.

## Discussion

The Irwin’s turtle (*E. irwini*) is a member of the snapping turtle genus of freshwater turtles endemic to Australia and Papua New Guinea [4, 38]. The *Elseya* complex are habitat specialists that need well-oxygenated waters with perennial flow to facilitate aquatic breathing [4], which limits their geographic range and habitat occupancy. A large proportion of the catchment where *E. irwini* is known to occur has been subject to water resource development and the species has not been seen in those reaches for > 25 years. The absence of current occurrence records is mainly due to the challenges to detect this species in their environment. The lack of information on the species biology, current distribution and population trends hinders it from being listed as threatened within state or federal law [4]. Here we conducted a catchment-wide eDNA assessment carried out by multiple sampling institutions to detect the presence of the iconic *E. irwini* in the Burdekin, Bowen and Broken Rivers, Queensland, Australia. We detected *E. irwini* DNA in the lower Burdekin at sites where contemporary evidence of turtle presence has long been absent. The highest proportion of positive eDNA detections occurred in the upper Bowen River and Broken River, most likely due to higher turtle densities in these areas. This study highlights the eDNA sampling methodology as a valid tool for detecting and monitoring this cryptic species in a standardised way without introducing bias due to different levels of expertise (or lack of) of the staff conducting fieldwork.

### Lower Burdekin River

This study constitutes the first scientific evidence of contemporary presence of *E. irwini* in the lower Burdekin River. Environmental DNA analysis of other aquatic organisms has also detected species presence within historical distributions where species were thought to be no longer present [39–41]. For example, eDNA sampling detected spotted gar at eight sites in the Lake Saint Clair catchment, Canada, where this species had not been recorded for over half a century, and the species was considered to have been locally extirpated [39]. Similarly, Sigsgaard et al. [42] used eDNA analysis to detect European weather loach at a historic site where the species had not been observed since 1995, and Janosik and Johnston [41] detected slackwater darter eDNA at a site where the species had not been found since the 1970s. Environmental DNA methods have also been used to locate over-wintering sites of freshwater turtles. Feng et al. [28] detected the northern map turtle eDNA from water samples collected under ice. Detections were then confirmed by visual surveys using remotely operated vehicles [28]. Similarly, Tarof et al. [25] detected eDNA of a threatened freshwater turtle at brumation sites with no previous records of species presence. The authors suggested that even during brumation, when turtles are not active or feeding, cloacal breathing would result in DNA shedding [25]. In the case of *E. irwini*, it is unknown whether eDNA detected in the present study originated from viable populations or a few older individuals that have persisted since dam construction.

Positive eDNA detections of *E. irwini* at four consecutive sites downstream from the Burdekin Falls Dam (sites 10–13 on Fig. 1) suggest the presence of a local population within this reach. These sites are above the junction with the Bowen River so cannot be the result of downstream transport of eDNA from that river, where there is known *E. irwini* contemporary presence. The Burdekin Falls Dam, built above a waterfall within a rocky gorge, is not historically known or considered to be habitat for *E. irwini*. Thus, our results provide strong evidence for an extant population of *E. irwini* within this river reach below that dam. We detected eDNA at two sites within the Gorge Weir, near the weir wall indicating that this species can persist in shallow weir impoundments that exhibit a high amount of suspended sediments, despite such conditions providing physiological constraints of these turtles [17]. We also detected *E. irwini* DNA at the junction of the Burdekin and Bowen Rivers, where the turtle was sighted during the early 1990s [4] and in 2004 (TropWATER unpublished data). This suggests remnant populations of *E. irwini* are still present at the river junction, where persistently turbid river waters from the Burdekin River join clear waters from the Bowen River.

The low percentage of eDNA detections of *E. irwini* and the fact that it was detected at scattered sites beyond the junction of the Burdekin and Bowen Rivers (sites 1–6 on Fig. 1) may indicate the presence of remnant populations along the lower Burdekin River especially at two sites (1—The Rocks; and 3—Millaroo) with positive eDNA detections that are likely too far downstream for the eDNA detected to be the result of downstream transport. After this work was conducted, we became aware of one *E. irwini* sighting at Dalbeg by campers (GPS points: 20.23575°S, 147.3104°E; Additional file 2: Fig. S2), approximately 4.7 km downstream from where our sampling site was located. The direct sighting of the species at this site support the hypothesis that the source of the positive eDNA detections at Dalbeg (albeit low) is live *E. irwini* present in the lower Burdkein River, rather than eDNA transported from upstream areas, where there were higher percentage of positive detections. Finally, we found inconsistencies in eDNA detection between two sites: Dalbeg and Johnny Cake Road (sites 5 and 6 on Fig. 1). These sites were adjacent, but water samples were collected by two different institutions. While two technical replicates amplified for eDNA at Dalbeg, no detections were observed at Johnny Cake Road. We attribute the inconsistencies to the small amount of eDNA present in the water, possibly due to the low eDNA shedding rate of the small pockets of remnant populations in the area. Inconsistent amplification of turtle eDNA can be explained by the stochastic nature of eDNA at low concentration in the presence of low density of individuals [26], rather than bias introduced by field staff.

### Bowen River

We detected eDNA at several sites (sites 22, 23, 24 on Fig. 1) in the middle reaches of the Bowen River. However, with the exception of one record of a juvenile immediately below the Collinsville Weir in 2009 and three females approximately 3.5 km downstream from that weir in 2012 (TropWATER unpublished data), these these sites have not been focus of surveys due to crocodile presence, therefore, our results provide new records. Here there are large, well-oxygenated clearwater pools that provide suitable habitat. However, these sites have not been the focus of in-water surveys for *E. irwini* due to the risk of crocodile attack, so this result provides new information on their distribution. There are historical and contemporary records of *E. irwini* at the Bowen/Burdekin junction itself (site 9 on Fig. 1) (Atlas of Living Australia, ala.org.au; Cann *pers. comm.*; TropWATER unpublished data). Also, a paratype of *E. irwini* was collected at the junction of Sandalwood (also referred to as Terrible Creek) and the Bowen River in 1994 (Queensland Museum, Q. M. J59021) [15]. Despite this, sampling sites in the lower Bowen River section did not detect the presence of *E. irwini* DNA. A recent eDNA study of a threatened freshwater turtle found high rates of type II error when attempting to detect the species at sites of known presence [25]. The authors found positive detections at 50% of their positive control sites and attributed this to suboptimal field replication [25]. In the present study, all five biological replicates were collected from different positions within a site to maximise the chances of capturing eDNA that may not be dispersed evenly in the environment [34]. Although the processed water volume has been successfully used to detect rare species in the past [37] there is a possibility that processing a larger volume of water could have detected eDNA present in low copy number. It is known that filtering high volumes of water can reduce the stochasticity of eDNA detection and this is why some authors recommend filtering as much water as it is possible [36]. However, in the present study system, water filtration would have comprised a time-consuming task impeding us to sample large areas in such a small timeframe (i.e., 12 sites sampled in  a single day). False negative detections found in this river could have also potentially arisen due to the amount of DNA insitu being under the LOD of the assay [43]. In a study testing eDNA detection of a recently eradicated fish, Furlan et al. [44] determined that the amount of samples required to detect the remnant fish in the lake were too large to make the study logistically and economically feasible. Although *E. irwini* has never been formally recorded from the vicinity of sites 15–21, it is known from above and below that reach and it would be hard to conclude that it does not occur in this reach. We therefore hypothesise that the lack of detections in the lower Bowen River is most likely due to very low turtle density.

### Broken River

The Broken River, especially its main tributary, Urannah and Massey Creek, have long been thought to be the core habitat of *E. irwini* [17]. However, survey effort (mainly snorkeling) has been heavily focused here because the water is clear and free from crocodiles, enabling efficient underwater visual census. Thus it is uncertain if the higher turtle densities recorded in this area are due to more favourable habitat or more favourable survey conditions. The present study illustrates that eDNA is found at a greater number of sites and in a greater proportion of technical replicates in the Broken River and Urannah Creek, confirming their presumed status as more favourable habitat for *E. irwini*.

Sites 35 and 36 (Fig. 1) were above the Eungella Dam and it is believed that the natural range of *E. irwini* does not extend that far upstream due to waterfalls that restrict upstream passage (TropWATER unpublished data). Also, sites 33 and 34 (the latter technically on the dry upper reaches of the Bowen River) are also considered to be outside the natural range of *E irwini*. Thus these eDNA results match with the known distribution of the species. It is notable that although known from tributaries in the upper reaches of the Broken River, *E. irwini* is known only from the main river channels of the Bowen and Burdekin rivers, not their tributaries. Outside of the Broken River, these tributaries are dry and have limited flow, providing unsuitable habitat for *E. irwini*. Thus the eDNA results for the main tributary creeks Expedition Pass Creek, Terrible Creek, Pelican Creek, Grant Creek and the drier upper Bowen River at Blenheim, all match the known or expected species distribution. One exception however, is Massey Creek. This rainforest-fed tributary of the Broken River has perennial flow and suitable pools, but very difficult access. Visual surveys have determined high *E. irwini* densities at a site located 4 km upstream from its junction with the Broken River (TropWATER *unpublished data*), on a farm property we were unable to access for this study. The single site we were able to access on Massey Creek for this study (site 33 on Fig. 1) was 5 km upstream of that site (9 km upstream from the junction with the Broken River), yet no eDNA was detected there. We hypothesize that our sampling site was too far upstream for *E. iriwni* to inhabit, being well within shallow, rainforest habitat. We suggest that further sampling is needed along Urannah and Massey Creek to determine the upstream limits of *E. irwini* distribution in these key tributaries. This is particularly important given the proposed Urannah Dam will inundate much of the permanent waterholes of Massey Creek and Urannah Creek, which our study confirms is a key habitat for *E. irwini*. The proposed Urannah Dam, which would be located on the Broken River just below the Urannah Creek junction, is a 970,000 megalitre, ~ 6100 hectare water storage that will provide water for a new irrigation precinct, pumped hydro-electric power storage and power generation and a water supply to nearby coal mines.

### Environmental DNA detectability in relation to water quality parameters

Despite the main covariates predicting detection of *E. irwini* DNA were dissolved oxygen and water conductivity, considerable uncertainty exists in the estimated occurrence probabilities of *E. irwini* eDNA, reflected in the large 95% confidence intervals. As mentioned before, this species relies on well-oxygenated waters to support its cloacal breathing [8, 13, 17]; therefore, it would have been expected to find dissolved oxygen driving eDNA detectability. However, evidence on the association between eDNA probability of detection and water quality parameters is not consistent. While Tarof et al. [25] found an association between eDNA probability of detection of a freshwater turtle and total dissolved solids (TDS) in the water, studies testing the extent of the effect of water quality parameters on fish and amphibian eDNA detection did not find water conductivity to influence eDNA detectability [34, 46]. Goldberg et al. [34] attributed their findings to the fact that the sampling systems had relatively even water conductivity values. In this study, we observed large variations between conductivity values across sites, which could be driving our results. It is also possible that the probability of eDNA detection of different taxa depends on different water quality parameters.

### Non-specialist engagement

The consistent results between sites sampled by JCU and BRU, as well as RDMW and BRU suggest that the field sampling protocol is robust and not biased by the expertise of the staff conducting the work. Also, no contamination was observed in field blanks, further supporting the idea that our easy-to-follow field method allows better sampling collection practices. The most widely accepted method of eDNA capture is water filtration. While we recognise that large volumes of water need to be processed in certain ecosystems, such as the ocean, to account for dilutionand water movement, we propose water precipitation in freshwater systems as a valid eDNA capture method. We have proven that precipitating eDNA from preserved whole water samples can yield consistent eDNA concentrations that can be detected via qPCR [37, 47]. Interestingly, the amount of water we sampled in the present study (300 mL per replicate) was larger than the the filtration volumes (90 mL and 250 mL) used by other published studies on freshwater turtle eDNA [31, 48]. In the present study, eDNA detection patterns at sites with known presence or abundance information, have largely concorded with each other, providing confidence for the results of sites surveyed only for eDNA.

## Conclusions

We successfully detected *E. irwini* eDNA in the lower section of the Burdekin River, Queensland, where the species has not been observed since the 1990s, thus indicating that a population of turtles persists in this region. We also confirmed that the upper reaches of the Broken River, including Urannah Creek are the core habitat of *E. irwini* but that consistent eDNA detections were also revealed in the poorly studied middle-upper Bowen River. While direct observations of *E. irwini* are needed to gather data on population size, age classes and female-male ratios (required for listing the species and developing management actions to protect the species), eDNA analysis provides valuable information on population distribution and potential changes over time. Environmental DNA has been suggested as the only efficient tool for rare and cryptic species detection [49]. In the case of *E. irwini* in the Burdekin River, we propose eDNA sampling as the most pragmatic detection method due to high water turbidity, the presence of crocodiles and the inability to reliably attract these turtles to traps. Finally, the simple steps of our sampling protocol allow any user to conduct eDNA sampling with minimal training, while avoiding sample contamination that could be introduced when carrying out water filtration in turbid waters.

## Methods

### Study system

*Elseya irwini* was discovered in 1990 at the junction of the Burdekin and Bowen Rivers, Queensland; however, there have not been any formal records of the species downstream of this location in the lower Burdekin River since specimens were collected by John Cann in 1993/1994 as part of its formal description in 1997 [15]. Other areas of the Burdekin River upstream of the Bowen/Burdekin river confluence but below the dam wall have never been surveyed ([4], TropWATER unpublished data). Contemporary occurrence records suggest the presence of *E. irwini* in the Bowen River and its major tributary—the Broken River (Atlas of Living Australia, ala.org.au; TropWATER unpublished data). Additionally, snorkelling surveys in the upper reaches of the Broken River, where the water is clear and well-oxygenated (and crocodiles are not present) suggest contemporary presence of *E. irwini* at high density (TropWATER *unpublished data*), especially near the Urannah Creek/Broken River junction. We, therefore, used sites at this location (Table 2) as positive control sites.

**Table 2:** Sampling sites for *E. irwini* eDNA detection along the Burdekin, Bowen and Broken River catchments

Catchment	Site name	Latitude (°S)	Longitude (°E)	Collection date	Site access	Sampling carried out by
Burdekin	The Rocks^a^ [1]	19.7036	147.2919	21/09/2020	Road	RDMW
	Bogie River^b^ [2]	20.0515	147.3643	16/12/2020	Helicopter	JCU
	Millaroo^a^ [3]	20.0552	147.2902	21/09/2020	Road	RDMW
	Expedition Pass Creek^b^ [4]	20.1512	147.2744	21/09/2020	Road	RDMW
	Dalbeg^a^ [5]	20.2775	147.3083	21/09/2020	Road	RDMW
	Johnny Cake Road^a^ [6]	20.2792	147.3103	28/04/2021	Road	BRU
	Burdekin and Bowen junction^a^ [7]	20.4041	147.3186	16/12/2020	Helicopter	JCU
	Blue Valley 1^a^ [8]	20.4125	147.3469	22/09/2020	Road	RDMW
	Blue Valley 2^a^ [9]	20.4042	147.3606	22/09/2020	Road	RDMW
	Gorge Weir 1^a,c^ [10]	20.4728	147.2922	22/09/2020	Road	RDMW
	Gorge Weir 2^a,c^ [11]	20.4736	147.2897	28/04/2021	Road	BRU
	Burdekin Falls Dam downstream 1^a^ [12]	20.5264	147.2837	16/12/2020	Helicopter	JCU
	Burdekin Falls Dam downstream 2^a^ [13]	20.5636	147.2372	16/12/2020	Helicopter	JCU
Bowen	Terrible Creek^a^ [14]	20.4522	147.3917	23/09/2020	Road	RDMW
	Terrible Creek upstream^a^ [15]	20.4573	147.4041	16/12/2020	Helicopter	JCU
	Riverview^a^ [16]	20.4597	147.5142	23/09/2020	Road	RDMW
	Bowen River Hotel^a^ [17]	20.5308	147.5563	23/09/2020	Road	RDMW
	Bowen River Hotel upstream^a^ [18]	20.5507	147.5625	16/12/2020	Helicopter	JCU
	Pelican Creek^b^ [19]	20.5539	147.6178	28/04/2021	Road	BRU
	Myuna 1^a^ [20]	20.5602	147.5864	16/12/2020	Helicopter	JCU
	Myuna 2^a^ [21]	20.5872	147.6043	16/12/2020	Helicopter	JCU
	Birralee^a^ [22]	20.6778	147.6719	23/09/2020	Road	RDMW
	Bowen Developmental Road^a^ [23]	20.7547	147.8553	24/09/2020	Road	RDMW
	Exmoor Road^a^ [24]	20.7489	147.9672	24/09/2020	Road	RDMW
Broken	Broken River^a^ [25]	20.8314	148.1140	16/12/2020	Helicopter	JCU
	Mount Sugarloaf^a^ [26]	20.8306	148.1786	16/12/2020	Helicopter	JCU
	Grant Creek^b^ [27]	20.8406	148.2649	29/04/2021	Road	BRU
	Urannah Creek 1^b^ [28]	20.9055	148.4377	29/04/2021	Road	BRU
	Urannah Creek 2^b^ [29]	20.9006	148.4381	16/12/2020	Helicopter	JCU
	Urannah Creek 3^b^ [30]	20.8952	148.4470	29/04/2021	Road	BRU
	Urannah Creek 4^b^ [31]	20.8891	148.4521	29/04/2021	Road	BRU
	Urannah Creek 5^b^ [32]	20.8973	148.4711	16/12/2020	Helicopter	JCU
	Massey Gorge^b^ [33]	21.0267	148.4200	30/04/2021	Road	BRU
	Blenheim^b^ [34]	21.0681	148.2122	24/09/2020	Road	RDMW
	Old Racecourse^b^ [35]	21.1939	148.4472	24/09/2020	Road	RDMW
	Resort^a^ [36]	21.1664	148.5014	24/09/2020	Road	RDMW
	Bee Creek^b^ (37)	21.1133	148.4522	30/04/2021	Road	BRU

Numbers in brackets next to site name indicate the site number in Fig. 1. Institutions carrying out field sampling were the Regional Development, Manufacturing and Water, Queensland (RDMW); James Cook University (JCU) and Bowen River Utilities (BRU)

^a^Sites located on the main river channel; ^b^sites located on a tributary; ^c^sites located on a weir

### Environmental DNA sampling

Five replicate water samples (biological replicates) were collected at each sampling site during three sampling events in 2020 and 2021 (Table 2). Since *E. irwini* inhabits deep pools with perennial water flow [4], sampling sites were selected by targeting water bodies with those characteristics along the Burdekin, Bowen and Broken Rivers and its tributaries, although in some tributary streams, we sampled at the only available sites with water present. The first sampling round (carried out by Queensland state government staff from the Department of Regional Development, Manufacturing and Water, RDMW) was conducted during September 2020, and comprised 16 sites accessible via road. A second sampling round was undertaken during December 2020 (carried out by James Cook University, JCU, scientists), where 12 further sites were surveyed. Those sites were located in more remote, upstream areas and were accessed via helicopter. Finally, a third sampling round was carried out during April 2021 by staff from the development company Bowen River Utilities (BRU), and it comprised nine sites that were accessed via road. The first and second sampling rounds were carried out in coordination between institutions (RDMW and JCU). However, the third sampling round was carried out independently by BRU as part of a consultancy project. Therefore, the sampling sites were selected without prior knowledge of the results from the two previous trips. Because of this, some sites occurred close to sites sampled by RDMW and JCU, opening an opportunity to test for consistency in eDNA detection when using the same field sampling protocols.

The field protocol for eDNA sample collection used in the present study has been developed at TropWATER JCU and consists of collecting whole water samples and directly preserving them in a non-alcohol based buffer [50]. The protocol instructions are contained in a four-page manual and require no prior face-to-face training [50]. This method has been used previously and showed reliable eDNA detection of a Critically Endangered rainforest frog > 20 km downstream from the species occurrence [37].Given that the target species inhabits the bottom of pools [4], water samples were collected from approximately 1.5–2 m below the water surface by attaching a new, clean plastic sampling jar of 500 mL capacity to an extension pole. Prior to this, the extension pole was rinsed three times by submerging it in the water and moving it side to side, downstream from the sampling area. Sampling consisted of collecting 300 mL of water using the aforementioned plastic jar and decanting it into another new, clean plastic jar (500 mL capacity) containing 100 mL Longmire’s preservative buffer [51]. The final volume of water sample and Longmire’s buffer was 400 mL. Additionally, one field blank was collected at each site which consisted of decanting 300 mL MilliQ water into a jar containing 100 mL Longmire’s buffer. All jars were kept in the dark in plastic crates at ambient temperature until arrival at the laboratory. It has been demonstrated that the Longmire’s buffer can keep eDNA in water samples intact for at least 3 months after collection when stored at tropical ambient temperature [47] and in Setrivex filters for up to 8 months [52]. During the second and third sampling rounds, data on water quality parameters were collected at each site, namely: temperature (°C), pH, dissolved oxygen (in mg L^−1^ and percentage) and conductivity (µS cm^−1^). Measurements were taken from sub-surface waters.

### Environmental DNA extractions

All eDNA extractions and qPCR analyses were carried out at the JCU-TropWATER dedicated eDNA laboratory. Upon returning to the laboratory, the exterior of all sampling jars was washed with 2% decon solution and blot dried. From each field replicate and controls we processed a total subsample of 100 mL water plus Longmire’s preservative buffer. To do this, five aliquots of 20 mL each were decanted into five DNA LoBind (Eppendorf®) Falcon tubes of 50 mL capacity. This approach has been previously used to detect eDNA of a Critically Endangered frog species in northern Australia 20 km downstream from where the species occurs [37]. Environmental DNA was extracted from samples using a glycogen-aided ethanol precipitation method [53]. Briefly, each 20 mL sample was mixed with 5 µL glycogen (20 mg/mL), 5 mL NaCl (5 M) and 20 mL ispropanol, vortexed and incubated at 4 °C overnight. Tubes were then centrifuged at 6,750 g for 10 min, the supernatant was discarded and the pellet was resuspended in 120 µL lysis buffer. The resuspended pellet from all five aliquots belonging to each field replicate were then pooled into a 2 mL DNA LoBind tube, constituting a total of 600 µL lysis buffer per field replicate. Samples were then kept at − 20 °C overnight. Subsequently, samples were thawed, vortexed at maximum speed for 30 s and incubated at 50 °C for five hours. At the end of this period, samples were allowed to come to room temperature, 1200 µL PEG-NaCl buffer and 1 µL glycogen was added and samples were stored at 4 °C overnight. Following this, tubes were centrifuged at 14,000 g for 30 min, and the pellet was washed twice with 70% ethanol. The pellet was air-dried and 100 µL TE buffer was added. Finally, a DNA purification step was performed using the DNeasy PowerClean Pro Clean up kit (Qiagen Pty. Ltd.) following the manufacturer’s protocol and samples were eluted in 100 µL elution buffer. For each eDNA extraction batch, an extraction control was added to ensure that no contamination was introduced during laboratory procedures [54].

### Real-time quantitative PCR (qPCR)

*Elseya irwini* detection was carried out using a species-specific eDNA assay developed and validated by TropWATER, targeting a 127 base pair (bp)-long fragment of the NADH dehydrogenase 4 (ND4) mitochondrial gene (Additional file 2). This eDNA assay can detect both *E. irwini* and an undescribed, closely related species from the Daintree area (far north Queensland), *E*. sp. Daintree (Additional file 2). Sanger sequencing of resulting amplicons can differentiate between both species (Additional file 2). The assay’s limit of detection (LOD) was determined to be 4.2 DNA copies/µL, while the limit of quantification (LOQ) was 420 DNA copies/µL (Additional file 2). All qPCR plates were set-up using the EzMate™ 401 Automated Pipetting System (Arise Biotech) and run in a QuantStudio™ 5 Real-Time PCR System (Thermo Fisher Scientific Australia Pty Ltd) using white 384-well plates sealed with optical films (Thermo Fisher Scientific Australia Pty Ltd). Eight technical replicates of each biological replicate at each site, including field and extraction blanks were tested. Additionally, each plate included three no-template controls, consisting of MilliQ water, and a triplicate positive control, consisting of *E. irwini* genomic DNA. Each qPCR assay consisted of 3 µL template DNA and 7 µL of master mix (5 µL Environmental Mastermix 2.0; 0.5 µL forward primer, 10 µM; 0.5 µL reverse primer, 10 µM; 0.5 µL TaqMan probe, 10 µM; 0.5 µL MilliQ water). Thermal cycling conditions were as follows: initial denaturation and activation at 95 °C for 10 min, then 50 cycles of 95 °C for 15 secs and 60 °C for 1 min. Inhibition was tested in most samples, including controls, using a TaqMan™ Exogenous Internal Positive Control (IPC) qPCR assay (ThermoFisher Scientific). A total of 1.5 µL IPC was applied to duplicate samples and three reactions containing only IPC were included as controls. A departure of 3 or more Ct cycles would indicate sample inhibition (Hartman et al. 2005). A subset of amplicons with positive detections were Sanger sequenced for confirmation of results at the Australian Genome Research Facility (AGRF).

### Occupancy modeling

Occupancy models were applied to *E.iriwni* eDNA detection data (Additional file 1: Table S3) and water quality parameters data (Additional file 1: Table S4) in order to determine whether environmental factors influence eDNA detectability. Note that site “Bogie River” was excluded from the analysis given that the measuring probe hit the bottom of the river when collecting water quality parameter data and unreliable records were obtained. The posterior probability of *E.irwini* eDNA detection and 95% confidence intervals (credible intervals) at each site were calculated using the eDNAoccupancy R package [55]. The eDNAoccupancy package uses a Bayesian approach that handles nested eDNA data [55]. A total of eight models were run testing different combinations of water quality parameters as explanatory variables for eDNA detection. We used dissolved oxygen as the explanatory variable for eDNA presence at a site, given that this water quality parameter is crucial for the turtle’s bimodal respiration [4]. The explanatory variables for eDNA presence in a sample and a qPCR technical replicate were water temperature, conductivity and pH, given that these factors have been repeatedly proven to affect eDNA detectability [34, 56–58]. Models were fitted using the *occModel* function, with MCMC chains run for 11,000 iterations, with 10,000 retained for parameter and confidence interval estimation. Competing models fitted to the data were then tested using the Watanabe-Akaike information criterion (WAIC) [59]. The model with the smallest WAIC value was selected to be the best performing [59].

## Supplementary Information

**Additional file 1.** Environmental DNA analysis information, including Δ Ct vales from qPCR internal positive controls (IPCs) (Table S1), as well as eDNA detection (Table S3) and survey data (Table S4) for occupancy models and models results (Table S2).

**Additional file 2.** Environmental DNA assay development and validation for *E. irwini* and *E.* sp. Daintree.

## Data Availability

All data supporting the findings of this study is under Additional files 1 and 2.
