# Supplementary material for: Environmental DNA analysis confirms extant populations of the cryptic Irwin’s turtle within its historical range
Source: BMC Ecol Evol. 2022 May 2;22:57. doi: 10.1186/s12862-022-02009-6 (PMC9059348; doi:10.1186/s12862-022-02009-6)
Supplement: Supplementary file 2 — Additional file 2. Environmental DNA assay development and validation for E. irwini and E. sp. Daintree. [file 12862_2022_2009_MOESM2_ESM.docx]

Environmental DNA analysis confirms extant populations of the cryptic Irwin's turtle within its historical range

Cecilia Villacorta-Rath^1^, Tom Espinoza^2^, Bernie Cockayne^2, 3^, Jason Schaffer^1^, Damien Burrows^1^

^1^Centre for Tropical Water and Aquatic Ecosystem Research (TropWATER), James Cook University, Townsville, QLD 4811, Australia

^2^ Department of Regional Development, Manufacturing and Water, Bundaberg, QLD, Australia

^3^ Reef Catchments (Mackay Whitsunday Isaac) Limited, Mackay, QLD, Australia

**Environmental DNA assay development and validation for *Elseya irwini* and *Elseya* sp. Daintree**

*Sequence data for primer development*

Given the genetic similarity between distinct populations of *E. irwini* occurring in the Burdekin catchment, the Johnstone River catchment and an undescribed *Elseya* species occurring in the Daintree River, Far North Queensland (1), denoted as *E.* sp. Daintree from now on, we developed and validated one eDNA assay targeting both species. Mitochondrial genetic sequences from the target and non-target species were searched and downloaded from the National Centre for Biotechnology Information (NCBI) database using Geneious Prime *version* 2020.1.2 (Table S1). Out of all the mitochondrial genes with available data, the NADH dehydrogenase 4 (ND4) gene showed the highest number of sequences for most of the Australian freshwater turtle species. Accessioned sequences were then aligned in Geneious Prime *version* 2020.1.2 and one probe-based assay was designed to amplify a 127-base pair (bp) section of the ND4 gene (Table S2). Dimerization of the candidate primer pair was tested using the software AutoDimer (2). No primer-dimer hits for the candidate primer pair were detected.

**Table S1.** List of target and non-target Australian freshwater turtle species tested *in-silico* for *E. irwini* primer development

| **Species** | **Accession number** |
| --- | --- |
| **Target species** |  |
| *Elseya irwini*  *Elseya* sp. Daintree | KC755114- KC755115  KF255948- KF255959 |
| **Non-target species** |  |
| *Carettochelys insculpta* | AY259596, AY673526 |
| *Chelodina (Chelodina) canni* | NC_041286; KY776448 |
| *Chelodina (Chelodina) longicollis* | KM581393-KM581420 |
| *Chelodina (Macrochelodina) expansa* | KJ469917-KJ469937 |
| *Elseya albagula* | JX871349-JX871353; KC755109 |
| *Elseya dentata* | KC755112-KC755113; KF255948-KF255950 |
| *Elseya lavarackorum* | KC755115; KF255952-KF255953 |
| *Elusor macrurus* | KC755124-KC755125 |
| *Emydura macquarii krefftii* | KF181854-KF181882 |
| *Emydura subglobosa subglobosa* | KC755127-KC755128 |
| *Emydura subglobosa worrelli* | KC755133 1; KC755133 2; KF255961 1; KF255961 2 |
| *Emydura tanybaraga* | KC755129-KC755130 |
| *Emydura victoriae* | KC755131-KC755131 |
| *Myuchelys bellii* | KC755134 |
| *Myuchelys georgesi* | KC755135 |
| *Myuchelys latisternum* | 41699843 |
| *Rheodytes leukops* | KC755137-KC755138; KF255962 |

**Table S2.** *Elseya* spp. assay information

**Primer/Probe Sequence 5’ – 3’ Fragment GC content Annealing**

**size (bp) (%) temp (°C)**

Espp_F AACTCAATCTGCCATACCCAAAGA 24 41.1 60.3

Espp_R TCTACATGTGCTTTTGGTAGTCACA 25 40.0 60.5

Espp_P CTCACACAACATGATGATTCGCCTT 25 44.0 62.1

*In-vitro assay specificity*

The candidate primer pair was tested *in-vitro* for specificity by conducting an end-point PCR using genomic DNA (gDNA) from target and non-target species as template. The target species tested included *E. irwini* specimens collected from the Burdekin (denoted as EI on Fig. S1) and Johnstone (denoted as ES on Fig. S1) River catchments, as well as *E.* sp. Daintree specimens (denoted as ESPD on Fig. S1) collected from the Daintree River. Non-target species tested included: *Elseya albagula*, *Elseya oneiros (formerly lavarackorum)*, *Elseya dentata, Myuchelys latisternum*, *Chelodina longicollis*, *Emydura tanybaraga*, *Emydura subglobosa worrelli*, *Emydura macquarii macquarii*, *Emydura macquarii kreftii*, *Emydura macquarii emmotti and Rheodytes leukops*. Out of these, the only co-occurring species are *Myuchelys latisternum* and *Emydura macquarii krefftii*.

A PCR was run on 20 µL reactions consisting of: 12.5 µL REDTaq ReadyMix (Sigma-Aldrich), 1 µL forward primer (10 µM), 1 µL reverse primer (10 µM), 3.5 µL MilliQ® water and 2 µL DNA template. The thermocycling conditions were as follows: initial denaturation at 94 °C for 1 min, followed by 35 cycles of annealing at 60 °C for 2 min and a final extension 72 °C for 3 min. Assay specificity was tested trough agarose gel electrophoresis using 2% agarose gels run at 65 V for 60 min. Agarose gels of PCR amplicons showed amplification of *E. irwini* and *E.* sp Daintree specimens from all three river catchments tested and no amplification of non-target species (Fig. S1).


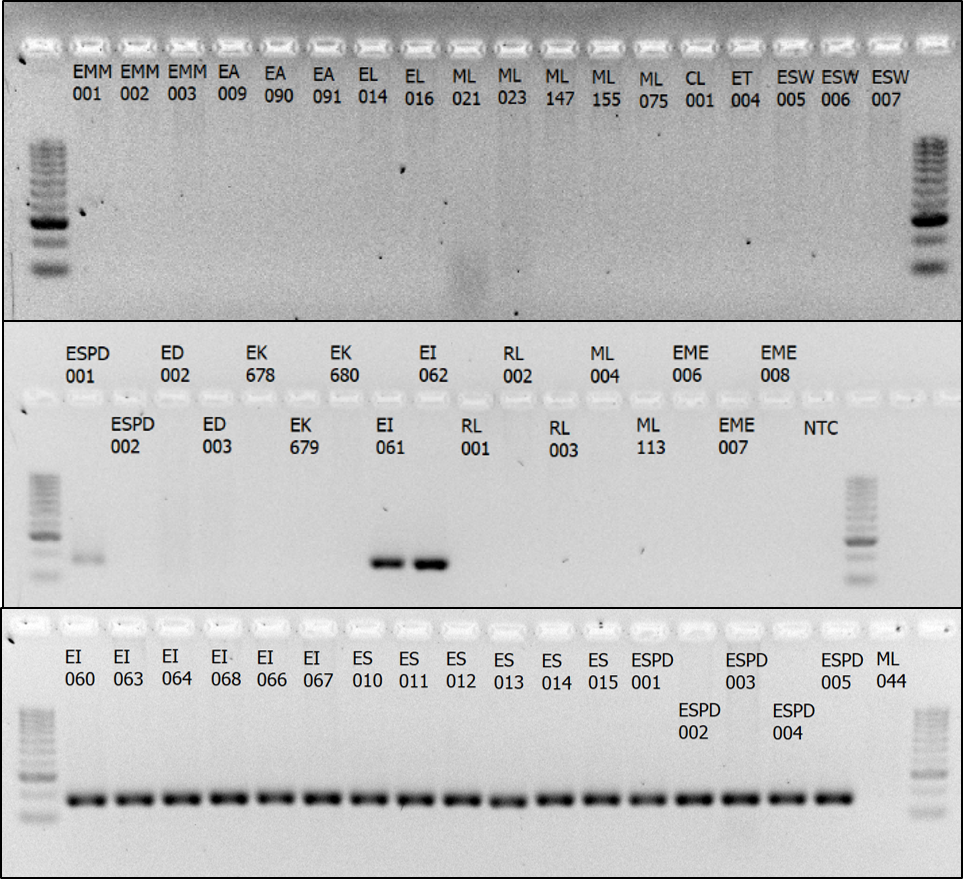


**Figure S1.** Test of candidate primer pair specificity on gDNA of target and non-target species. Bands of approximately 127 bp length were only visible for *E. irwini* and *E.* sp Daintree gDNA. Species codes: EMM (*Emydura macquarii macquarii*); EA (*Elseya albagula*); EL (*Elseya oneiros*, former *Elseya lavarackorum*); ML (*Myuchelys latisternum*); CL (*Chelodina longicollis*); ET (*Emydura tanybaraga*); ESW (*Emydura subglobosa worrelli*); ESPD (*Elseya* sp. Daintree); ED (*Elseya dentata*); EK (*Emydura macquarii kreftii*); EI (*Elseya irwini*, Burdekin River catchment); RL (*Rheodytes leukops*); EME (*Emydura macquarii emmotti*); ES (*Elseya irwini*, Johnstone River catchment); ESPD (*Elseya* sp. Daintree); NTC (no template control)

Following this, qPCR amplification of gDNA of the target species and two co-occurring species was carried out in 10 µL reactions consisting of: 5 µL Environmental Master Mix 2.0 (Thermo Fisher); 0.5 µL forward primer (10 µM); 0.5 µL reverse primer (10 µM); 0.5 µL probe (10 µM); 0.5 µL MilliQ water; and 3 µL gDNA template. A 2-step qPCR was run under the following thermocycling conditions: 95°C for 10 min followed by 45 cycles of 95°C for 15 s and 60°C for 1 min. qPCR plates were set-up manually in white 96-well plates and run in a QuantStudio 3 Real-Time PCR System (Thermo Fisher Scientific Australia, Pty Ltd). Positive amplification of target gDNA was observed.

*Limit of detection*

The limit of detection (LOD) and limit of quantification (LOQ) were estimated using a 12-fold serial dilution of double-stranded synthetic DNA fragments (gBlocks^TM^ Integrated DNA Technologies Pty Ltd, New South Wales, Australia) synthesized to match the target fragments of the target species. Serial dilutions ranged between 4 x 10^7^ DNA copies/µL and 0.001 DNA copies/µL. Twenty-four technical replicates of each dilution were run under a QuantStudio™ 5 Real-Time PCR System (Thermo Fisher Scientific, Australia Pty Ltd) using the same thermocycling conditions as above in a white 384-well plate. The LOD was set at the lowest standard with 95% or greater detection following the method outlined by Klymus et al. (3). Based on the serial dilutions, the LOD was determined to be 4.2 DNA copies/µL and the LOQ was 420 DNA copies/µL.

*Field validation*

Water samples for *E. irwini* and *E.* sp. Daintree eDNA assay validation were collected from three sites on the Johnstone River and three sites in the Daintree River, respectively, during June 2020 (Table S3), where these species are known to occur. Each field replicate (biological replicate) consisted of 300 mL of water mixed with 100 mL Longmire’s preservative buffer and kept at ambient temperature until arriving to the laboratory.

**Table S3.** Field sites where water was collected for field validation of *E.* spp. assay along the Johnstone and Daintree Rivers

**Location Site Latitude Longitude Sampling date**

Daintree River Site 1 16°10’51’’ 145°17’00’’ 29/06/2020

Site 2 16°10’34’’ 145°16’41’’ 29/06/2020

Site 3 16°11’55’’ 145°17’31’’ 29/06/2020

Johnstone River Warracker Creek 17°31’19’’ 145°54’43’’ 30/06/2020

Fisher Creek 17°33’53’’ 145°54’23’’ 30/06/2020

Paronella Park 17°39’11’’ 145°57’26’’ 30/06/2020

Environmental DNA was extracted in the TropWATER–JCU eDNA laboratory upon return to the laboratory. A 100 mL aliquot of sample plus Longmire’s buffer of each field replicate (including field controls) was decanted into five Falcon tubes (50 mL capacity) for eDNA extractions. We used a glycogen-aided precipitation method as described in the main text of the manuscript. qPCR reactions were set as specified in section “*In-vitro* assay specificity”. Positive detections were Sanger sequenced to confirm specificity of the assay at the Australian Genome Research Facility (AGRF).

All sampling sites exhibited positive *E. irwini* and *E.* sp eDNA detections. Positive *E. irwini* eDNA detections matched the ND4 gene of the target species (accession number NC041282) at 100% pairwise identity between positions 10,765–10,892 bp. Additionally, positive *E.* sp. Daintree eDNA detections matched the ND4 gene of the target species (accession numbers KF255958 or KF255959) at 100% pairwise identity between positions 176–299 bp.

**Table S4.** Results of *E. irwini* and *E.* sp. Daintree eDNA detections at three sites along two rivers where the target species are known to occur

| **Location** | **Site** | **Biological replicates** | | | **Technical replicates** | | |
| --- | --- | --- | --- | --- | --- | --- | --- |
|  |  | **# reps** | **# pos reps** | **% pos detections** | **# reps** | **# pos reps** | **% pos detections** |
| **Daintree River** | **1** | 5 | 2 | 40 | 20 | 2 | 10 |
|  | **2** | 5 | 3 | 60 | 20 | 3 | 15 |
|  | **3** | 5 | 4 | 80 | 20 | 7 | 35 |
| **Johnstone River** | **Warracker Creek** | 5 | 4 | 80 | 20 | 10 | 50 |
|  | **Fisher Creek** | 5 | 4 | 80 | 20 | 5 | 25 |
|  | **Paronella Park** | 5 | 3 | 60 | 20 | 7 | 35 |

**Table S5.** Checklist of Minimum Information for Publication of Quantitative Real-Time PCR Experiments (MIQE)(4). Essential and desirable information are included

| **Item to check** |  | **Information** |
| --- | --- | --- |
| Experimental design | Definition of experimental and control groups | Experimental: (1) genomic DNA (gDNA) from the target species |
|  |  | (2) environmental DNA (eDNA) samples consisting of water from |
|  |  | pond/creek/river where target species are known to occur |
|  |  | Control: MilliQ water |
|  | Number within each group | gDNA: n *E. irwini* = 14; n *E.* sp Daintree = 5; eDNA: n = 15 per species |
| Sample (gDNA) | Description | Samples were collected from multiple drainages per species to investigate intraspecific lineage diversity. Tissues were sourced from existing collections, through collaborations with other researchers, or during targeted fieldwork (1). |
|  | Microdissection or macrodissection | Small sections of skin were removed from the hindlimbs of turtles (1) |
|  | Processing procedure: |  |
|  | If fixed – with what, how quickly? | Tissue samples were preserved immediately in 95% ethanol (1) |
|  | Sample storage condition and duration (especially for FFPE samples) | Extracted DNA was stored at -20°C (1) |
| Sample (eDNA) | Description | Direct collection and preservation of 300 mL water from water body where the species was known to occur |
|  | Processing procedure | Water samples were split into five aliquots of 20 mL each (100 mL total) for eDNA extraction using the PPLPP method (5) |
|  | Sample storage condition and duration (especially for FFPE samples) | Extracted DNA was stored at 4°C for one week (until qPCR was performed) and subsequently stored at -20°C |
| Nucleic acid extraction (gDNA) | Procedure and/or instrumentation: |  |
|  | Name of kit and details of any modifications | Modified salting-out protocol described by Todd et al. (2013) |
|  | Details of DNase or RNase | N/A |
|  | Contamination assessment (DNA or RNA) | Nanodrop |
|  | Nucleic acid quantification |  |
|  | Instrument and method | Quantus ONE kit |
|  | RNA integrity method/instrument |  |
|  | RIN/RQI or Cq of 3' and 5' transcripts | N/A |
|  | Inhibition testing (Cq dilutions, spike or other) | N/A |
| Nucleic acid extraction (eDNA) | Procedure and/or instrumentation |  |
|  | Name of kit and details of any modifications | PPLPP method (5) with a subsequent purification using the DNeasy PowerClean CleanUp Kit (Qiagen®) |
|  | Details of DNase or RNase | N/A |
|  | Contamination assessment (DNA or RNA) | Nanodrop |
|  | Nucleic acid quantification |  |
|  | Instrument and method | N/A |
|  | RNA integrity method/instrument |  |
|  | RIN/RQI or Cq of 3' and 5' transcripts | N/A |
|  | Inhibition testing (Cq dilutions, spike or other) |  |
| qPCR target information | Gene symbol | N/A |
|  | Sequence accession number | *E. irwini*: NC041282; *E.* sp Daintree: KF255958, KF255959 |
|  | Amplicon length | 127 bp |
|  | *In silico* specificity Screen (BLAST, etc.) | BLAST |
|  | Location of each primer and probe by exon or intron (if applicable) |  |
|  | What splice variants are targeted? | N/A |
|  | Primer and probe sequences | Forward primer: 5'–AACTCAATCTGCCATACCCAAAGA–3' |
|  |  | Reverse primer: 5'–TCTACATGTGCTTTTGGTAGTCACA–3' |
|  |  | Probe: 5'–FAM–CTCACACAACATGATGATTCGCCTT–3' |
|  | Location and identity of any modifications | NADH dehydrogenase 4 (ND4) region of the mitochondrial genome |
|  | Complete reaction conditions: |  |
|  | Reaction volume and amount of cDNA/DNA | Reaction volume: 10 μL; amount of DNA: 3 μL |
| qPCR oligonucleotides | Primer, probe, Mg^2+^ and dNTP concentrations | Each 10 µL reaction contained: 5 µL Environmental master Mix 2.0; 0.5 µL forward primer (10 µM); 0.5 µL reverse primer (10 µM); 0.5 µL probe (10 µM); 0.5 µL MilliQ water; 3 µL template DNA |
|  | Polymerase identity and concentration | Applied Biosystems® TaqMan® Environmental Master Mix 2.0 (Thermo Fisher Scientific) |
|  |  |  |
| qPCR protocol | Buffer/kit identity and manufacturer | Applied Biosystems® TaqMan® Environmental Master Mix 2.0 (Thermo Fisher Scientific) |
|  | Additives (SYBR Green I, DMSO, etc.) | N/A |
|  | Complete thermocycling parameters | 95°C for 10 min followed by 45 cycles of 95°C for 15 s and 60°C for 1 min |
|  | Manufacturer of qPCR instrument | QuantStudio5 (Applied Biosystems) |
|  | Specificity using genomic DNA (gel, sequence, melt or digest) | Gel - Figure S1, Supplementary Material 2 |
|  | Specificity using eDNA samples (gel, sequence, melt or digest) | Specificity was confirmed by direct amplicon sequencing (dual direction Sanger sequencing) of eDNA samples |
|  | For SYBR Green I, Cq of the NTC | N/A |
| qPCR validation | Standard curves with slope and y–intercept | Slope: -3.573; y–intercept: 37.5 |
|  | PCR efficiency calculated from slope | 90.48% |
|  | R^2^ of standard curve | 0.762 |
|  | Linear dynamic range | 2–420000 DNA copies/reaction |
|  | Cq variation at lower limit | Cq mean: 35.6883; Cq Standard deviation: 1.1512; Cq coefficient of variation: 0.9436 |
|  | Evidence for LOD | LOD was set at the lowest standard with 95% or greater detection (following method in Klymus et al. (3)) |
|  | If multiplex, efficiency and LOD of each assay | N/A |
|  | qPCR analysis program (source, version) | QuantStudio Design & Analysis Software (Agilent Technologies, Inc.) |
|  | Cq method determination | Following method from Klymus et al. (3) |
|  | Outlier identification and disposition | Following method from Klymus et al. (3) |
|  | Results of NTCs | No amplification |
| Data analysis | Justification of number and choice of reference genes | N/A |
|  | Description of normalisation method | Standard curve |
|  | Number and stage (RT or qPCR) of technical replicates | Four technical qPCR replicates of each biological replicate |
|  | Repeatability (intra-assay variation) | N/A |
|  | Statistical methods for result significance | Klymus et al. (3) |
|  | Software (source, version) | R Statistical software |

**Figure S2.** *Elseya irwini* specimen found by campers at “Dalbeg” site, lower Burdekin River, where there were eDNA detections. Photo: McShane family

**
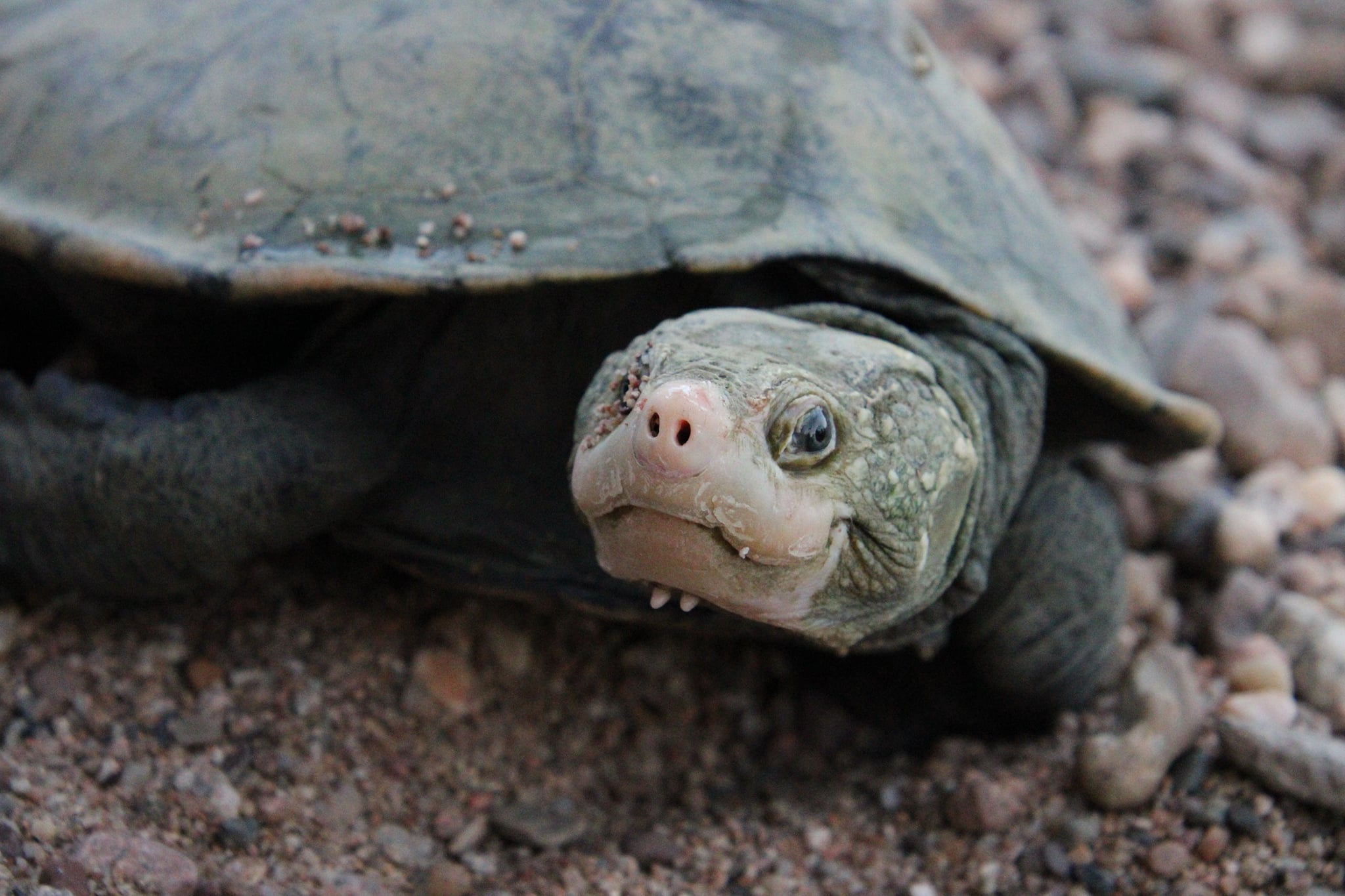
**

**References**

1. Todd E V., Blair D, Georges A, Lukoschek V, Jerry DR. A biogeographical history and timeline for the evolution of Australian snapping turtles (Elseya: Chelidae) in Australia and New Guinea. J Biogeogr. 2014;41(5):905–18.

2. Vallone PM, Butler JM. AutoDimer: A screening tool for primer-dimer and hairpin structures. Biotechniques. 2004;37(2):226–31.

3. Klymus KE, Merkes CM, Allison MJ, Goldberg CS, Helbing CC, Hunter ME, et al. Reporting the limits of detection and quantification for environmental DNA assays. Environ DNA. 2019;(May):1–12.

4. Bustin SA, Benes V, Garson JA, Hellemans J, Huggett J, Kubista M, et al. The MIQE guidelines: Minimum information for publication of quantitative real-time PCR experiments. Clin Chem. 2009;55(4):611–22.

5. Edmunds RC, Burrows DW. Got glycogen?: Development and multi-species validation of the novel Preserve, Precipitate, Lyse, Precipitate, Purify (PPLPP) workflow for environmental DNA extraction from Longmire’s preserved water samples. J Biomol Tech. 2020;31(4):125–50.

6. Todd E V., Blair D, Farley S, Farrington L, Fitzsimmons NN, Georges A, et al. Contemporary genetic structure reflects historical drainage isolation in an Australian snapping turtle, Elseya albagula. Zool J Linn Soc. 2013;169(1):200–14.
